# The crystal structures of 3-*O*-benzyl-1,2-*O*-iso­propyl­idene-5-*O*-methane­sulfonyl-6-*O*-tri­phenyl­methyl-α-d-gluco­furan­ose and its azide displacement product

**DOI:** 10.1107/S205698901800765X

**Published:** 2018-05-31

**Authors:** Zane Clarke, Evan Barnes, Kate L. Prichard, Laura J. Mares, Jack K. Clegg, Adam McCluskey, Todd A. Houston, Michela I. Simone

**Affiliations:** a Discipline of Chemistry, University of Newcastle, Callaghan, NSW 2308, Australia; b Juniata College, Department of Chemistry, 1700 Moore Street, Huntingdon, Pennsylvania, PA16652-2196, USA; c Priority Research Centre for Chemical Biology & Clinical Pharmacology, University of Newcastle, Callaghan, NSW 2308, Australia; d School of Chemistry and Molecular Biosciences, University of Queensland, Brisbane St Lucia, QLD 4072, Australia; e Institute for Glycomics and The School of Environment and Science, Griffith University, Gold Coast Campus, Southport, QLD 4222, Australia

**Keywords:** crystal structure, imino­sugar, d-gluco­furan­ose, elimination, substitution, hydrogen bonding, C—H⋯π inter­actions

## Abstract

The effect of different leaving groups on the substitution *versus* elimination outcomes with C-5 d-glucose derivatives was investigated. The stereochemical configurations of 3-*O*-benzyl-1,2-*O*-iso­propyl­idene-5-*O*-methane­sulfonyl-6-*O*-tri­phenyl­methyl-α-d-gluco­furan­ose, C_36_H_38_O_8_S (**3**) [systematic name: 1-[(3a*R*,5*R*,6*S*,6a*R*)-6-benz­yloxy-2,2-di­methyl­tetra­hydro­furo[2,3-*d*][1,3]dioxol-5-yl)-2-(trit­yloxy)ethyl methane­sulfonate], a stable inter­mediate, and 5-azido-3-*O*-benzyl-5-de­oxy-1,2-*O*-iso­propyl­idene-6-*O*-tri­phenyl­methyl-β-l-ido­furan­ose, C_35_H_35_N_3_O_5_ (**4**) [systematic name: (3a*R*,5*S*,6*S*,6a*R*)-5-[1-azido-2-(trit­yloxy)eth­yl]-6-benz­yloxy-2,2-di­methyl­tetra­hydro­furo[2,3-*d*][1,3]dioxole], a substitution product, were examined and the inversion of configuration for the azido group on C-5 in **4** was confirmed. The absolute structures of the mol­ecules in the crystals of both compounds were confirmed by resonant scattering. In the crystal of **3**, neighbouring mol­ecules are linked by C—H⋯O hydrogen bonds, forming chains along the *b*-axis direction. The chains are linked by C—H⋯π inter­actions, forming layers parallel to the *ab* plane. In the crystal of **4**, mol­ecules are also linked by C—H⋯O hydrogen bonds, forming this time helices along the *a*-axis direction. The helices are linked by a number of C—H⋯π inter­actions, forming a supra­molecular framework.

## Abstract 2

The effect of different leaving groups on the substitution *versus* elimination outcomes with C-5 d-glucose derivatives was investigated.

## Chemical context   

**Figure 1:**
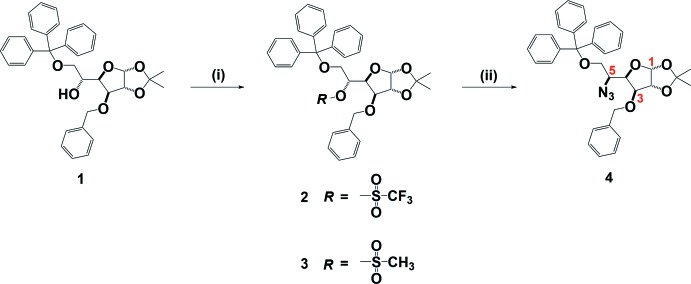
The synthesis of the title compounds. Reagents and conditions. (i) tri­fluoro­methane­sulfonyl anhydride, DCM, pyridine, 243 K (to **2**); methane­sulfonyl chloride, DMAP, DCM, Et_3_N (67%) (to **3**); (ii) NaN_3_, DMF, r.t. (88% over two steps from **1** *via* **2**). The numbering system used is highlighted in red.

Nucleophilic substitution reactions and their competition with elimination are mechanistically complex processes in carbohydrate systems (Latham *et al.*, 2017 ▸; Monnier *et al.*, 2008 ▸; Kroh *et al.*, 2008 ▸; Hayase *et al.*, 2002 ▸; Jin *et al.*, 2008 ▸; Chheda *et al.*, 2007 ▸; Reza *et al.*, 2014 ▸; Srokol *et al.*, 2004 ▸; Chuntanapum & Matsumura, 2010 ▸; Stemann *et al.*, 2013 ▸). Leaving groups that are normally readily displaced by substitution in simple carbon scaffolds can react to give mixtures of substitution (both with retention and inversion of configuration) and elimination products in monosaccharides and derivatives thereof (Latham *et al.*, 2017 ▸; Tsuchiya *et al.*, 1985 ▸; Tsuchiya, 1990 ▸; Mulard *et al.*, 1994 ▸; Hasegawa *et al.*, 1985 ▸; Karpiesiuk *et al.*, 1989 ▸; Yamashita *et al.*, 1984 ▸; Vos *et al.*, 1984 ▸). Introduction of a leaving group at position C-5 of d-glucose derivative **1** (Fig. 1 ▸) provides a potential opportunity for specific nucleophilic substitutions (*e.g*. with azide) or installation of a C=C moiety. Elimination gives rise to four possible alkenes *via* cis and/or *trans* isomers with either a C-4/C-5 or a C-5/C-6 disposed double bond. Prior reports suggest that the latter pathway is more probable (Gramera *et al.*, 1964*a* ▸,*b* ▸; Buchanan & Oakes, 1965 ▸).

In our development of novel imino­sugars (Simone *et al.*, 2012 ▸; Soengas *et al.*, 2012 ▸; Reed *et al.*, 2013 ▸), we viewed the installation of a C-5 disposed double bond (through elimination) and the ability to stereoselectively substitute at C-5 (through substitution) as critical to analogue development. To effect these transformations in an orthogonal manner (Fig. 1 ▸), we probed the nature of the C-5 leaving group through the introduction of a mesylate (**2**) and a triflate moiety (**3**), which could then be either displaced or eliminated. We had previously noted that C-6 OH silylation (TES, TBDMS, TIPS) afforded a high degree of protecting-group lability; as such, this moiety was trityl protected. With analogues **2** and **3** in hand, treatment with sodium azide under S_N_2 conditions afforded substituted azido product **4** in 88% yield. To confirm the stereochemistry of the starting triflate/mesylate (**2** and **3**) and azide **4**, these analogues were carefully crystallized. Mesylate **3** was crystallized by diffusion from CH_2_Cl_2_/hexane to give colourless, block-like crystals while azide **4** was readily crystallized from an ethanol/toluene mixture affording large, colourless crystals. Reaction conditions to afford the regioselective elimination product/s with a C-4/C-5 and/or a C-5/C-6 disposed double bond are currently under investigation.
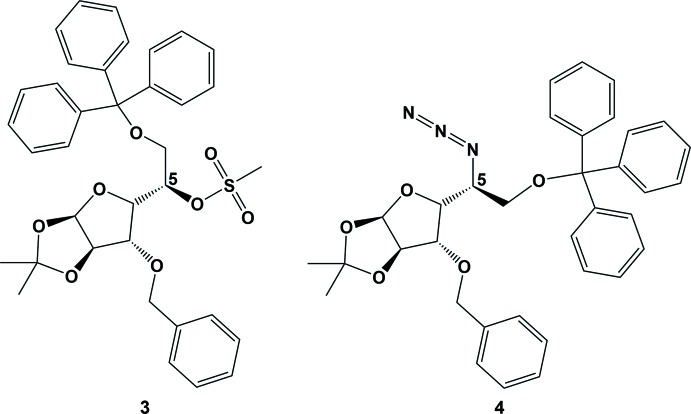


## Structural commentary   

**Figure 2:**
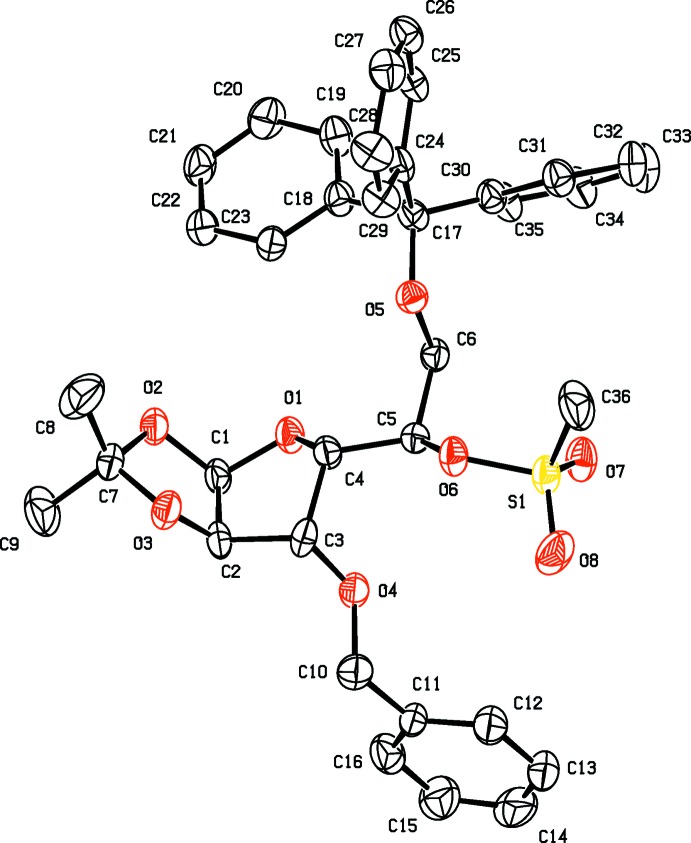
A view of the mol­ecular structure of compound **3**, with atom labelling and displacement ellipsoids drawn at the 30% probability level. For clarity, H atoms have been omitted.

The mol­ecular structures of compounds **3** and **4** are illustrated in Figs. 2 ▸ and 3 ▸, respectively. Notable, and anti­cipated, is the inversion of configuration for the azido group on C5 in compound **4**.

In **3** the central tetra­hydro­furan (THF) ring (O1/C1–C4) has a twisted conformation on the C3—C4 bond, with quasi-axial departure of the benzyl group from C3 and in the opposite direction of the iso­propyl­idene group from C1 and C2 (Fig. 2 ▸). This conformation accommodates the sterically bulky trityl moiety, which projects equatorially from C4. The 2,2-dimethyl-1,3-dioxolane ring (O2/O3/C1/C2/C7) also has a twisted conformation, on the O3—C7 bond, and its mean plane is inclined to the mean plane of the THF ring by 65.6 (7)°.

**Figure 3:**
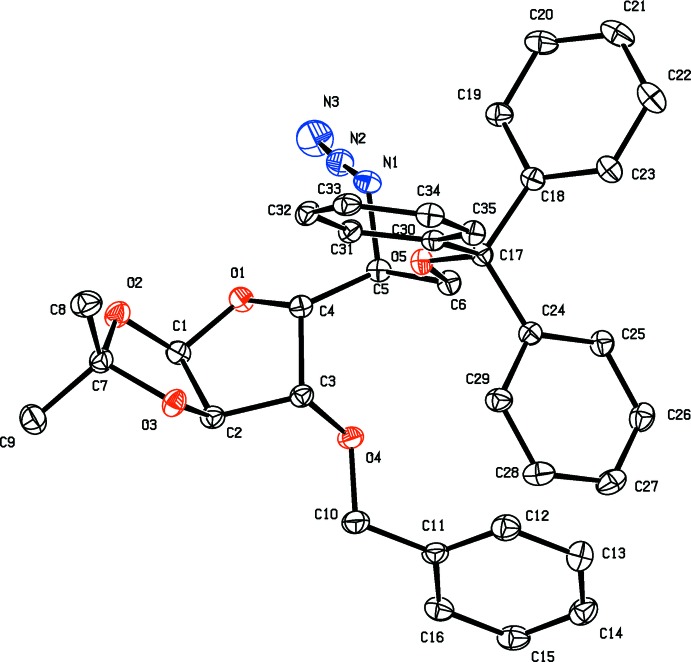
A view of the mol­ecular structure of compound **4**, with atom labelling and displacement ellipsoids drawn at the 30% probability level. For clarity, H atoms have been omitted.

The X-ray structure analysis of **4** shows that the THF ring has an envelope conformation with atom C4 as the flap. The pendant bonds adopt a conformation highly similar to that observed for **3** (Fig. 3 ▸). As in **3**, the 2,2-dimethyl-1,3-dioxolane ring has a twisted conformation on the O3—C7 bond, and its mean plane is inclined to the mean plane of the THF ring by 66.21 (9)°. The benzyl group is involved in a C—H⋯π inter­action with a phenyl ring of the tri­phenyl­methyl moiety, C12—H12⋯*Cg*5 (see Table 2 ▸ for details). The middle nitro­gen atom of the azide, which is cationic, appears to be involved in a weak ion–dipole intra­molecular inter­action with the endocyclic THF oxygen atom [N2⋯O1 = 2.900 (2) Å].

## Supra­molecular features   

**Figure 4:**
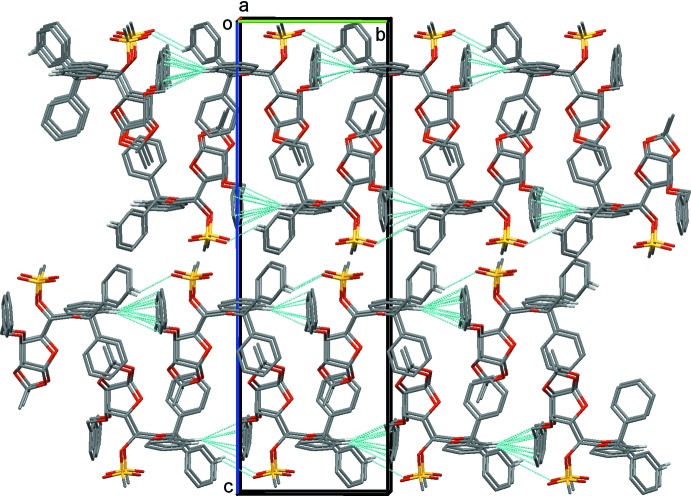
A view along the *a* axis of the crystal packing of compound **3**. The C—H⋯O and C—H⋯π inter­actions (see Table 1 ▸) are shown as dashed lines. For clarity, only the H atoms involved in these inter­actions have been included.

In the crystal of **3**, mol­ecules are linked by C—H⋯O hydrogen bonds, forming chains propagating along the *b*-axis direction (Table 1 ▸). The chains are linked by C—H⋯π inter­actions, so forming layers lying parallel to the *ab* plane (Table 1 ▸ and Fig. 4 ▸).

**Figure 5:**
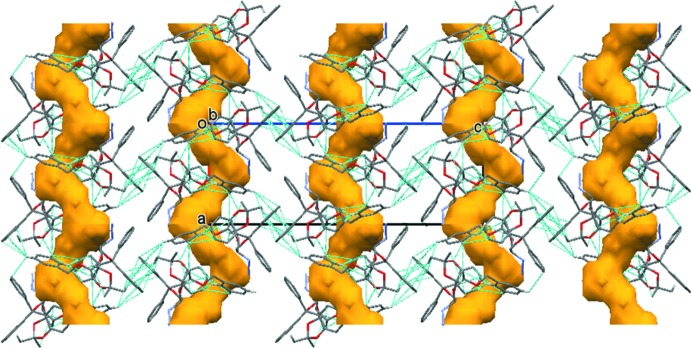
A view along the *b* axis of the crystal packing of compound **4**. The C—H⋯O and C—H⋯π inter­actions (see Table 2 ▸) are shown as dashed lines. For clarity, only the H atoms involved in these inter­actions have been included. The channels in the crystal structure are shown in brown (*Mercury*; Macrae *et al.*, 2008 ▸).

In the crystal of **4**, mol­ecules are also linked by C—H⋯O hydrogen bonds, forming 2_1_ helices propagating along the *a*-axis direction (Table 2 ▸). The helices are linked by a number of C—H⋯π inter­actions, so forming a supra­molecular framework (Table 2 ▸ and Fig. 5 ▸). In the crystal, there are voids with a potential solvent-accessible volume of *ca* 161 Å^3^ (5% of the unit-cell volume). However, on examination of the final difference-Fourier map no evidence could be found of electron density being present in the channels.

## Database survey   

Sulfonate esters (*e.g*. tri­fluoro­methane­sulfonates, *para*-toluene­sulfonates and methane­sulfonates) make up an important class of inter­mediates in organic chemistry for their role as leaving groups in nucleophilic substitutions. X-ray crystallographic analyses of sulfonate esters are limited by the degree of their chemical instability. Relative solvolysis rates for tri­fluoro­methane­sulfonates, *para*-toluene­sulfonates and methane­sulfonates are, respectively, in the ranges 1.4 × 10^8^, 3.7 × 10^4^, and 3.0 × 10^3^ compared to chloride (Noyce &Virgilio, 1972 ▸). Notable literature examples of monosaccharide-derived sulfonate ester crystal structural studies include the only reported primary tri­fluoro­methane­sulfonate (Simone *et al.*, 2007 ▸), primary and secondary *para*-toluene­sulfonates (Reed *et al.*, 2013 ▸; Mamat *et al.*, 2012 ▸), primary and secondary mesylates (Krajewski *et al.*, 1992 ▸; Sofian *et al.*, 2002 ▸), dimesylates (Adiwidjaja *et al.*, 2000 ▸; Armishaw *et al.*, 1996 ▸; Brown *et al.*, 1986 ▸; Craythorne *et al.*, 2009 ▸) and a trimesylate (Voss *et al.*, 2016 ▸).

## Synthesis and crystallization   

The reagents and conditions used for the syntheses of compounds **3** and **4** are outlined in Fig. 1 ▸. Reactions were performed under an atmosphere of nitro­gen gas and maintained using an inflated balloon. Further general experimental details are included in the archived CIF.

**Synthesis of compound 3:** 3-*O*-benzyl-1,2-*O*-iso­propyl­idene-6-*O*-tri­phenyl­methyl-α-d-gluco­furan­ose **1** (520 mg, 0.943 mmol) was dissolved in CH_2_Cl_2_ (12 ml) with pyridine (260 µL, 3.262 mmol) and 4-di­methyl­amino­pyridine (40 mg, 0.327 mmol). Methane­sulfonyl chloride (180 µL, 2.325 mmol) was added and the reaction mixture heated to reflux for 25 h. Thin layer chromatographic (TLC) analysis (1:4 ethyl acetate/hexa­ne) revealed complete consumption of the starting material (*R*_f_ = 0.46) and formation of the desired product (*R*_f_ = 0.52). The reaction mixture was pre-absorbed on silica gel and compond **3** was isolated by flash chromatography to give an off-white foam (449 mg, 76%) and recrystallized from CH_2_Cl_2_/hexa­nes yielding colourless block-shaped crystals [m.p. 397–403 K (433–434 K; Saeki *et al.*, 1968 ▸)]; [α]_D_^20^: −15.5° (*c* 0.11 in CHCl_3_; ^1^H NMR (400 MHz, CDCl_3_) δ 7.50–7.20 (*m*, 20 H, ArHs), 5.88 (*d*, 1H, *J*_H1,H2_ 3.6 Hz, H-1), 5.30 (ddd, 1H, *J*_H-5,H-4_ 8.4 Hz, *J*_H-5,H-6_ 6.0 Hz, *J*_H-5,H-6′_ 2.0 Hz H-5), 4.75 (*d*, 1H, *J*_BnCH,BnCH′_ 10.8 Hz, BnC*H*H′), 4.60–4.55 (*m*, 2H, H-2 & BnCH*H′*), 4.51 (*dd*, 1H, *J*_H-4,H-5_ 8.8 Hz, *J*_H-4,H-3_ 3.2 Hz, H-4), 4.14 (*d*, 1H, *J*_H-3,H-4_ 2.8 Hz, H-3), 3.65 (*dd*, 1H, *J*_H-6′,H-6_ 11.2 Hz, *J*_H-6′,H-5_ 2.0 Hz, H-6′), 3.44 (*dd*, 1H, *J*_H-6,H-6′_ 11.2 Hz, *J*_H-6,H-5_ 6.0 Hz, H-6), 2.89 (*s*, 3H, MsCH_3_), 1.35, 1.29 (2 × *s*, 2 × CH_3_ acetonide); ^13^C-NMR (100 MHz, CDCl_3_) δ 143.4 (Cquat trit­yl), 137.4 (ArCquat Bn), 128.7–127.2 (22 × ArC trityl, Bn), 112.1 (Cquat acetonide), 105.4 (C1), 87.0 (ArCquat trit­yl) 81.5 (C2), 81.2 (C3), 77.9 (C5), 77.8 (C4), 72.5 (BnCH_2_), 63.1 (C6), 39.3 (MsCH_3_), 26.8, 26.3 (2 × CH_3_ acetonide); ν_max_ (thin film): 2935, 2924, 2852 (*m*/*s*, ArCH and alkyl CH), 1461 (*m*, S=O), 1270 (*m*, alkyl aryl ether C—O), 1073 (*m*, S=O); HRMS *m*/*z* calculated for C_36_H_38_KO_8_S [*M* + K^+^]^+^ 669.19190 (100%), found 669.19148 (100%).

**Synthesis of compound 4:** 3-*O*-benzyl-1,2-*O*-iso­propyl­idene-6-*O*-tri­phenyl­methyl-α-d-gluco­furan­ose **1** (1.00 g, 1.81 mmol) was dissolved in CH_2_Cl_2_ (20 ml) and cooled to 243 K. Pyridine (291 µL, 3.62 mmol) was added and stirred for 10 min. Tri­fluoro­methane­sulfonic anhydride (607 µL, 3.62 mmol) was added dropwise with continued stirring. TLC analysis (1:4 ethyl acetate/hexa­nes) after 45 min showed complete consumption of the starting material (*R_f_* = 0.42) and formation of a new product (*R_f_* = 0.67). The reaction mixture was acidified with glacial acetic acid (5 ml) and washed with brine (3 × 20 ml). The organic layer was concentrated *in* vacuo and dissolved in *N*,*N*-di­methyl­formamide (25 ml). The solution was cooled to 243 K and sodium azide (345 mg, 5.31 mmol) was added. The reaction mixture was left to warm up to room temperature while stirring for 12 h. Analysis by TLC (1:4 ethyl acetate/hexa­ne) showed complete consumption of the triflate inter­mediate (*R*_f_ = 0.67) and formation of product (*R*_f_ = 0.38). Lithium chloride solution (30 ml, 5% *w*/*v*) was added followed by extraction with CH_2_Cl_2_ (3 × 30 ml). The combined organic layers were dried over sodium sulfate and concentrated *in* vacuo. The product, compound **4**, was recrystallized from chloro­form and ethanol yielding colourless prismatic crystals (917 mg, 88%). [α]_D_^26^ −15.8° (*c* 0.90 in CHCl_3_) [Lit. [α]_D_ −20.7° (*c* 1.06, DCM) (García-Moreno *et al.*, 2007 ▸)]; m.p. 441–444 K; ^1^H NMR (500 MHz, CDCl_3_) δ 7.47–7.00 (*m*, 20 H, ArHs), 5.93 (*d*, 1H, *J*_H-1,H-2_ 3.7 Hz, H-1), 4.49 (*d*, 1H, *J*_H-2,H-1_ 3.8 Hz, H-2), 4.47 (*dd*, 1H, *J*_H-4,H-5_ 9.0 Hz, *J*_H-4,H-3_ 3.2 Hz, H-4), 4.27 (*d*, 1H, *J*_BnCH,BnCH′_ 11.2 Hz, BnC*H*H′), 3.85 (ddd, 1 H, *J*_H-5,H-4_ 8.8 Hz, *J*_H-5,H-6_; 5.0 Hz, *J*_H-5,H-6_ 2.5 Hz, H-5), 3.75 (*d*, 1 H, *J*_BnCH′,BnCH_ 11.2 Hz, BnCH*H*′), 3.53 (*d*, 1 H, *J*_H-3,H-4_ 3.1 Hz, H-3), 3.43 (*dd*, 1 H, *J*_H-6,H-6′_ 9.7 Hz, J_H-6,H-5_ 2.3 Hz, H-6), 3.06 (*dd*, 1 H, *J*_H-6′,H-6_ 9.7 Hz, *J*_H-6′,H-5_ 5.1 Hz, H-6′), 1.53, 1.30 (2 × s, 2 ×CH_3_ acetonide); ^13^C NMR (125 MHz, CDCl_3_) δ 143.6 (C_quat_ trit­yl), 137.1 (ArC_quat_ Bn), 128.9–127.4 (22 × ArC), 112.1 (C_quat_ acetonide), 105.1 (C1), 87.0 (ArC_quat_ trit­yl), 82.5 (C3), 82.0 (C2), 79.9 (C4), 72.0 (BnCH_2_), 63.2 (C6), 61.5 (C5), 27.0, 26.6 (2 × CH_3_ acetonide); ν_max_ (thin film): 3061 (*w*, ArCH), 2986 (*m*, alkyl CH), 2097 (*s*, N_3_); HRMS (ESI–FT–ICR) *m*/*z* calculated for C_35_H_35_N_3_NaO_5_ [*M* + Na]^+^ 600.24689 (100%), found 600.24679 (100%).

## Refinement   

Crystal data, data collection and structure refinement details are summarized in Table 3 ▸. For both compounds, the H atoms were included in calculated positions and treated as riding: C—H = 0.95-1.00 Å with *U*_iso_(H) = 1.5*U*_eq_(C-meth­yl) and 1.2*U*_eq_(C) for other H atoms. The methane­sulfonyl group suffers from thermal disorder but attempts to split all S, O and C atoms did not significantly improve the refined structure.

## Supplementary Material

Crystal structure: contains datablock(s) 3, 4, global. DOI: 10.1107/S205698901800765X/su5437sup1.cif

Structure factors: contains datablock(s) 3. DOI: 10.1107/S205698901800765X/su54373sup3.hkl

Structure factors: contains datablock(s) 4. DOI: 10.1107/S205698901800765X/su54374sup2.hkl

CCDC references: 1844798, 1844797

Additional supporting information:  crystallographic information; 3D view; checkCIF report

## Figures and Tables

**Table 1:** Hydrogen-bond geometry (Å, °) for **3** [Chem scheme1] *Cg*3 is the centroid of the C11–C16 ring.

*D*—H⋯*A*	*D*—H	H⋯*A*	*D*⋯*A*	*D*—H⋯*A*
C34—H34⋯O8^i^	0.95	2.55	3.179 (17)	124
C25—H25⋯*Cg*3^ii^	0.95	2.99	3.830 (12)	149

Symmetry codes: (i) 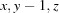; (ii) 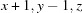.

**Table 2:** Hydrogen-bond geometry (Å, °) for **4** [Chem scheme1] *Cg*5 and *Cg*6 are the centroids of the C24–C29 and C30–C35 rings.

*D*—H⋯*A*	*D*—H	H⋯*A*	*D*⋯*A*	*D*—H⋯*A*
C13—H13⋯O3^i^	0.95	2.56	3.281 (2)	133
C1—H1⋯*Cg*5^ii^	1.00	2.90	3.8014 (16)	150
C8—H8*C*⋯*Cg*6^iii^	0.98	2.91	3.5435 (18)	123
C12—H12⋯*Cg*5	0.95	2.88	3.7503 (18)	153
C15—H15⋯*Cg*6^i^	0.95	2.91	3.6050 (18)	131

Symmetry codes: (i) 
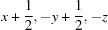
; (ii) 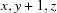; (iii) 
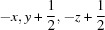
.

**Table 3:** Experimental details

	**3**	**4**
Crystal data		
Chemical formula	C_36_H_38_O_8_S	C_35_H_35_N_3_O_5_
*M*_r_	630.72	577.66
Crystal system, space group	Orthorhombic, *P*2_1_2_1_2_1_	Orthorhombic, *P*2_1_2_1_2_1_
Temperature (K)	190	150
*a*, *b*, *c* (Å)	10.0069 (5), 10.1898 (7), 32.0045 (14)	10.0943 (1), 10.9625 (1), 27.5392 (2)
*V* (Å^3^)	3263.4 (3)	3047.45 (5)
*Z*	4	4
Radiation type	Cu *K*α	Cu *K*α
μ (mm^−1^)	1.31	0.68
Crystal size (mm)	0.3 × 0.25 × 0.2	0.26 × 0.15 × 0.13

Data collection		
Diffractometer	Rigaku Xcalibur, Sapphire3, Gemini ultra	Agilent SuperNova, Dual, Cu at zero, Atlas
Absorption correction	Multi-scan (*CrysAlis PRO*; Rigaku OD, 2015 ▸)	Multi-scan (*CrysAlis PRO*; Rigaku OD, 2015 ▸)
*T*_min_, *T*_max_	0.840, 1.000	0.676, 1.000
No. of measured, independent and observed [*I* > 2σ(*I*)] reflections	20834, 5928, 4263	73112, 6389, 6282
*R*_int_	0.068	0.038
(sin θ/λ)_max_ (Å^−1^)	0.601	0.631

Refinement		
*R*[*F* ^2^ > 2σ(*F* ^2^)], *wR*(*F* ^2^), *S*	0.094, 0.351, 1.20	0.029, 0.085, 1.03
No. of reflections	5928	6389
No. of parameters	410	391
H-atom treatment	H-atom parameters constrained	H-atom parameters constrained
Δρ_max_, Δρ_min_ (e Å^−3^)	0.53, −1.07	0.39, −0.19
Absolute structure	Flack *x* determined using 1367 quotients [(*I* ^+^)−(*I* ^−^)]/[(*I* ^+^)+(*I* ^−^)] (Parsons *et al.*, 2013 ▸)	Flack *x* determined using 2717 quotients [(*I* ^+^)−(*I* ^−^)]/[(*I* ^+^)+(*I* ^−^)] (Parsons *et al.*, 2013 ▸)
Absolute structure parameter	0.004 (10)	−0.02 (3)

Computer programs: *CrysAlis PRO* (Rigaku OD, 2015 ▸), *SHELXT2014/7* (Sheldrick, 2015*a* ▸), *SHELXL2018/3* (Sheldrick, 2015*b* ▸), *SIR97* (Altomare *et al.*, 1999 ▸), *PLATON* (Spek, 2009 ▸), *Mercury* (Macrae *et al.*, 2008 ▸) and *publCIF* (Westrip, 2010 ▸).

## supplementary crystallographic information

### 1-[(3aR,5R,6S,6aR)-6-Benzyloxy-2,2-dimethyltetrahydrofuro[2,3-d][1,3]dioxol-5-yl]-2-(trityloxy)ethyl methanesulfonate (3) Crystal data

**Table:**

C_36_H_38_O_8_S	*D*_x_ = 1.284 Mg m^−^^3^
*M**_r_* = 630.72	Cu *K*α radiation, λ = 1.54184 Å
Orthorhombic, *P*2_1_2_1_2_1_	Cell parameters from 10052 reflections
*a* = 10.0069 (5) Å	θ = 4.5–70.0°
*b* = 10.1898 (7) Å	µ = 1.31 mm^−^^1^
*c* = 32.0045 (14) Å	*T* = 190 K
*V* = 3263.4 (3) Å^3^	Block, colourless
*Z* = 4	0.3 × 0.25 × 0.2 mm
*F*(000) = 1336	

### 1-[(3aR,5R,6S,6aR)-6-Benzyloxy-2,2-dimethyltetrahydrofuro[2,3-d][1,3]dioxol-5-yl]-2-(trityloxy)ethyl methanesulfonate (3) Data collection

**Table:**

Rigaku Xcalibur, Sapphire3, Gemini ultra diffractometer	4263 reflections with *I* > 2σ(*I*)
Radiation source: fine-focus sealed X-ray tube, Enhance Ultra (Cu) X-ray Source	*R*_int_ = 0.068
ω scans	θ_max_ = 68.0°, θ_min_ = 4.6°
Absorption correction: multi-scan (CrysAlis PRO; Rigaku OD, 2015)	*h* = −11→12
*T*_min_ = 0.840, *T*_max_ = 1.000	*k* = −12→12
20834 measured reflections	*l* = −33→38
5928 independent reflections	

### 1-[(3aR,5R,6S,6aR)-6-Benzyloxy-2,2-dimethyltetrahydrofuro[2,3-d][1,3]dioxol-5-yl]-2-(trityloxy)ethyl methanesulfonate (3) Refinement

**Table:**

Refinement on *F*^2^	Hydrogen site location: inferred from neighbouring sites
Least-squares matrix: full	H-atom parameters constrained
*R*[*F*^2^ > 2σ(*F*^2^)] = 0.094	*w* = 1/[σ^2^(*F*_o_^2^) + (0.1748*P*)^2^ + 6.0087*P*] where *P* = (*F*_o_^2^ + 2*F*_c_^2^)/3
*wR*(*F*^2^) = 0.351	(Δ/σ)_max_ < 0.001
*S* = 1.20	Δρ_max_ = 0.53 e Å^−^^3^
5928 reflections	Δρ_min_ = −1.07 e Å^−^^3^
410 parameters	Extinction correction: (SHELXL-2018/3; Sheldrick 2015b), Fc^*^=kFc[1+0.001xFc^2^λ^3^/sin(2θ)]^-1/4^
0 restraints	Extinction coefficient: 0.0044 (11)
Primary atom site location: structure-invariant direct methods	Absolute structure: Flack *x* determined using 1367 quotients [(*I*^+^)-(*I*^-^)]/[(*I*^+^)+(*I*^-^)] (Parsons *et al.*, 2013)
Secondary atom site location: difference Fourier map	Absolute structure parameter: 0.004 (10)

### 1-[(3aR,5R,6S,6aR)-6-Benzyloxy-2,2-dimethyltetrahydrofuro[2,3-d][1,3]dioxol-5-yl]-2-(trityloxy)ethyl methanesulfonate (3) Special details

**Table:**

Geometry. All esds (except the esd in the dihedral angle between two l.s. planes) are estimated using the full covariance matrix. The cell esds are taken into account individually in the estimation of esds in distances, angles and torsion angles; correlations between esds in cell parameters are only used when they are defined by crystal symmetry. An approximate (isotropic) treatment of cell esds is used for estimating esds involving l.s. planes.

### 1-[(3aR,5R,6S,6aR)-6-Benzyloxy-2,2-dimethyltetrahydrofuro[2,3-d][1,3]dioxol-5-yl]-2-(trityloxy)ethyl methanesulfonate (3) Fractional atomic coordinates and isotropic or equivalent isotropic displacement parameters (Å^2^)

**Table:**

	*x*	*y*	*z*	*U*_iso_*/*U*_eq_	
S1	0.5662 (3)	0.7854 (3)	0.46165 (7)	0.0576 (8)	
O1	0.5510 (8)	0.6791 (7)	0.31101 (19)	0.0566 (19)	
O2	0.6330 (10)	0.7335 (8)	0.2462 (2)	0.067 (2)	
O3	0.6539 (9)	0.9404 (8)	0.2696 (2)	0.065 (2)	
O4	0.4019 (8)	0.8917 (7)	0.3505 (2)	0.0550 (18)	
O5	0.7705 (7)	0.5716 (7)	0.3878 (2)	0.0485 (16)	
O6	0.6203 (9)	0.7925 (8)	0.4151 (2)	0.063 (2)	
O7	0.4808 (9)	0.6727 (8)	0.4660 (2)	0.068 (2)	
O8	0.5109 (13)	0.9099 (9)	0.4710 (3)	0.094 (4)	
C1	0.5280 (14)	0.7491 (10)	0.2743 (3)	0.059 (3)	
H1	0.440997	0.723237	0.261309	0.071*	
C2	0.5292 (13)	0.8954 (11)	0.2854 (3)	0.058 (3)	
H2	0.450815	0.944503	0.273966	0.069*	
C3	0.5350 (13)	0.8960 (11)	0.3333 (3)	0.055 (3)	
H3	0.587619	0.971936	0.344310	0.067*	
C4	0.6029 (13)	0.7660 (10)	0.3421 (3)	0.053 (3)	
H4	0.701972	0.774736	0.339075	0.064*	
C5	0.5684 (13)	0.7023 (10)	0.3837 (3)	0.053 (2)	
H5	0.468940	0.698638	0.386460	0.063*	
C6	0.6231 (10)	0.5655 (10)	0.3885 (3)	0.047 (2)	
H6A	0.592268	0.527018	0.415221	0.056*	
H6B	0.590666	0.509275	0.365389	0.056*	
C7	0.6830 (14)	0.8585 (12)	0.2349 (3)	0.064 (3)	
C8	0.8334 (18)	0.850 (2)	0.2300 (6)	0.110 (6)	
H8A	0.855493	0.777777	0.210871	0.166*	
H8B	0.874231	0.832793	0.257338	0.166*	
H8C	0.867575	0.932418	0.218739	0.166*	
C9	0.613 (2)	0.9036 (15)	0.1955 (4)	0.094 (5)	
H9A	0.641165	0.993113	0.188667	0.141*	
H9B	0.515871	0.902173	0.199947	0.141*	
H9C	0.635774	0.844726	0.172374	0.141*	
C10	0.3419 (15)	1.0193 (11)	0.3553 (4)	0.065 (3)	
H10A	0.332671	1.062316	0.327718	0.077*	
H10B	0.398722	1.075298	0.373260	0.077*	
C11	0.2042 (14)	1.0014 (11)	0.3753 (3)	0.061 (3)	
C12	0.1939 (17)	1.0081 (14)	0.4184 (4)	0.073 (4)	
H12	0.270576	1.021812	0.435373	0.088*	
C13	0.067 (2)	0.9940 (17)	0.4362 (5)	0.103 (6)	
H13	0.056333	0.998517	0.465700	0.123*	
C14	−0.043 (2)	0.9736 (17)	0.4108 (7)	0.104 (6)	
H14	−0.128897	0.965806	0.423079	0.125*	
C15	−0.0304 (19)	0.9644 (15)	0.3689 (5)	0.091 (5)	
H15	−0.106376	0.948442	0.351806	0.109*	
C16	0.0966 (17)	0.9787 (13)	0.3510 (4)	0.078 (4)	
H16	0.106929	0.972430	0.321586	0.093*	
C17	0.8331 (11)	0.4437 (10)	0.3839 (3)	0.048 (2)	
C18	0.8224 (12)	0.3951 (11)	0.3388 (3)	0.054 (3)	
C19	0.8348 (13)	0.2635 (11)	0.3285 (3)	0.061 (3)	
H19	0.844259	0.200026	0.350075	0.074*	
C20	0.8336 (15)	0.2237 (14)	0.2869 (4)	0.073 (3)	
H20	0.844730	0.133531	0.280225	0.088*	
C21	0.8166 (15)	0.3136 (14)	0.2555 (3)	0.073 (4)	
H21	0.812525	0.285769	0.227217	0.087*	
C22	0.8054 (14)	0.4445 (13)	0.2654 (3)	0.065 (3)	
H22	0.795842	0.507915	0.243796	0.078*	
C23	0.8081 (12)	0.4834 (13)	0.3065 (3)	0.059 (3)	
H23	0.799835	0.574119	0.312841	0.071*	
C24	0.9853 (12)	0.4693 (9)	0.3914 (3)	0.049 (2)	
C25	1.0686 (12)	0.3622 (10)	0.3967 (3)	0.054 (3)	
H25	1.033200	0.275944	0.394830	0.065*	
C26	1.2063 (13)	0.3806 (12)	0.4050 (3)	0.061 (3)	
H26	1.263470	0.307176	0.408860	0.073*	
C27	1.2568 (14)	0.5071 (14)	0.4073 (4)	0.068 (3)	
H27	1.348633	0.520765	0.413357	0.081*	
C28	1.1724 (15)	0.6145 (13)	0.4007 (4)	0.074 (3)	
H28	1.207834	0.701004	0.401258	0.089*	
C29	1.0347 (13)	0.5951 (12)	0.3931 (4)	0.063 (3)	
H29	0.976890	0.668086	0.389262	0.075*	
C30	0.7800 (13)	0.3519 (11)	0.4171 (3)	0.056 (3)	
C31	0.8290 (14)	0.3567 (11)	0.4578 (3)	0.062 (3)	
H31	0.899595	0.415396	0.464453	0.074*	
C32	0.7762 (16)	0.2769 (14)	0.4887 (3)	0.073 (4)	
H32	0.812262	0.281411	0.516140	0.088*	
C33	0.6711 (16)	0.1897 (14)	0.4807 (4)	0.078 (4)	
H33	0.636079	0.134670	0.501964	0.093*	
C34	0.6204 (16)	0.1868 (14)	0.4406 (4)	0.075 (4)	
H34	0.548702	0.128922	0.434386	0.090*	
C35	0.6703 (13)	0.2649 (11)	0.4096 (3)	0.062 (3)	
H35	0.631387	0.261348	0.382575	0.075*	
C36	0.7142 (14)	0.763 (2)	0.4884 (5)	0.115 (7)	
H36A	0.696895	0.766101	0.518575	0.173*	
H36B	0.777068	0.833003	0.480916	0.173*	
H36C	0.752616	0.677798	0.481073	0.173*	

### 1-[(3aR,5R,6S,6aR)-6-Benzyloxy-2,2-dimethyltetrahydrofuro[2,3-d][1,3]dioxol-5-yl]-2-(trityloxy)ethyl methanesulfonate (3) Atomic displacement parameters (Å^2^)

**Table:**

	*U*^11^	*U*^22^	*U*^33^	*U*^12^	*U*^13^	*U*^23^
S1	0.0813 (18)	0.0600 (15)	0.0316 (11)	−0.0056 (14)	0.0007 (12)	−0.0015 (10)
O1	0.084 (5)	0.053 (4)	0.033 (3)	0.001 (4)	0.000 (3)	0.001 (3)
O2	0.100 (6)	0.057 (4)	0.044 (4)	0.005 (4)	0.019 (4)	0.002 (3)
O3	0.087 (6)	0.060 (4)	0.050 (4)	−0.006 (4)	0.024 (4)	0.004 (3)
O4	0.077 (5)	0.047 (4)	0.041 (3)	0.004 (4)	0.007 (3)	0.002 (3)
O5	0.052 (4)	0.052 (4)	0.042 (3)	0.004 (3)	−0.001 (3)	0.001 (3)
O6	0.094 (6)	0.063 (4)	0.031 (3)	−0.007 (4)	0.000 (3)	0.003 (3)
O7	0.097 (6)	0.061 (4)	0.046 (4)	−0.024 (4)	0.012 (4)	0.002 (3)
O8	0.145 (10)	0.067 (5)	0.072 (6)	0.009 (6)	0.041 (6)	−0.010 (4)
C1	0.090 (8)	0.055 (6)	0.032 (4)	−0.001 (6)	−0.007 (5)	0.002 (4)
C2	0.082 (8)	0.063 (6)	0.029 (4)	0.000 (6)	0.004 (5)	0.006 (4)
C3	0.082 (8)	0.052 (6)	0.032 (4)	−0.002 (6)	0.011 (5)	0.000 (4)
C4	0.077 (7)	0.052 (6)	0.031 (4)	0.003 (5)	−0.001 (4)	0.005 (4)
C5	0.078 (7)	0.048 (5)	0.032 (4)	0.015 (5)	−0.003 (5)	0.002 (4)
C6	0.048 (5)	0.057 (6)	0.036 (4)	−0.002 (4)	0.005 (4)	0.006 (4)
C7	0.078 (8)	0.069 (7)	0.044 (5)	0.006 (6)	0.023 (5)	0.005 (5)
C8	0.095 (12)	0.132 (15)	0.104 (12)	0.016 (12)	0.041 (10)	−0.009 (11)
C9	0.149 (15)	0.087 (10)	0.047 (6)	0.030 (10)	0.023 (8)	0.012 (6)
C10	0.094 (9)	0.045 (6)	0.055 (6)	0.003 (6)	0.003 (6)	−0.004 (5)
C11	0.087 (9)	0.051 (6)	0.044 (5)	0.010 (6)	0.019 (6)	0.000 (5)
C12	0.097 (10)	0.071 (8)	0.053 (6)	0.012 (7)	0.012 (7)	0.003 (6)
C13	0.145 (17)	0.094 (11)	0.069 (8)	0.038 (12)	0.053 (11)	0.025 (8)
C14	0.097 (13)	0.088 (11)	0.127 (14)	0.010 (10)	0.055 (12)	0.030 (10)
C15	0.101 (12)	0.070 (9)	0.102 (11)	−0.003 (8)	0.024 (10)	0.008 (8)
C16	0.113 (12)	0.068 (8)	0.052 (6)	0.019 (8)	−0.003 (7)	−0.004 (6)
C17	0.060 (6)	0.049 (5)	0.035 (4)	0.003 (5)	−0.009 (4)	0.004 (4)
C18	0.068 (7)	0.060 (6)	0.033 (4)	−0.007 (5)	−0.001 (4)	−0.001 (4)
C19	0.081 (8)	0.058 (7)	0.044 (5)	−0.007 (6)	−0.003 (5)	0.001 (5)
C20	0.096 (10)	0.069 (7)	0.054 (6)	−0.005 (7)	−0.004 (6)	−0.020 (6)
C21	0.084 (9)	0.095 (10)	0.039 (5)	0.001 (8)	−0.008 (6)	−0.011 (6)
C22	0.078 (8)	0.079 (8)	0.038 (5)	0.019 (7)	−0.006 (5)	−0.002 (5)
C23	0.067 (7)	0.070 (7)	0.040 (5)	0.008 (6)	0.007 (5)	0.003 (5)
C24	0.070 (7)	0.045 (5)	0.031 (4)	−0.005 (5)	0.005 (4)	0.006 (4)
C25	0.076 (7)	0.048 (5)	0.039 (5)	−0.005 (5)	−0.009 (5)	0.005 (4)
C26	0.078 (8)	0.069 (7)	0.037 (5)	−0.001 (6)	−0.009 (5)	0.003 (5)
C27	0.069 (8)	0.082 (8)	0.053 (6)	−0.009 (7)	−0.010 (6)	0.002 (6)
C28	0.085 (9)	0.060 (7)	0.076 (8)	−0.011 (7)	−0.011 (7)	−0.002 (6)
C29	0.069 (7)	0.057 (6)	0.063 (7)	−0.007 (6)	−0.011 (6)	0.000 (5)
C30	0.083 (8)	0.049 (6)	0.036 (5)	0.003 (5)	0.001 (5)	−0.002 (4)
C31	0.086 (8)	0.059 (6)	0.040 (5)	−0.005 (6)	−0.001 (5)	0.004 (5)
C32	0.102 (10)	0.083 (8)	0.035 (5)	−0.010 (8)	0.002 (6)	0.004 (5)
C33	0.101 (11)	0.084 (9)	0.048 (6)	−0.022 (8)	0.010 (6)	0.008 (6)
C34	0.092 (10)	0.076 (8)	0.055 (6)	−0.011 (7)	−0.008 (6)	0.011 (6)
C35	0.080 (8)	0.058 (6)	0.050 (5)	−0.016 (6)	−0.018 (5)	0.010 (5)
C36	0.055 (8)	0.22 (2)	0.075 (9)	−0.033 (11)	−0.040 (7)	0.051 (12)

### 1-[(3aR,5R,6S,6aR)-6-Benzyloxy-2,2-dimethyltetrahydrofuro[2,3-d][1,3]dioxol-5-yl]-2-(trityloxy)ethyl methanesulfonate (3) Geometric parameters (Å, º)

**Table:**

S1—O8	1.416 (10)	C14—C15	1.35 (2)
S1—O7	1.438 (8)	C14—H14	0.9500
S1—O6	1.587 (7)	C15—C16	1.40 (2)
S1—C36	1.726 (12)	C15—H15	0.9500
O1—C1	1.394 (11)	C16—H16	0.9500
O1—C4	1.429 (12)	C17—C30	1.512 (14)
O2—C1	1.392 (14)	C17—C18	1.528 (13)
O2—C7	1.415 (14)	C17—C24	1.564 (16)
O3—C7	1.419 (13)	C18—C23	1.378 (15)
O3—C2	1.423 (14)	C18—C19	1.386 (16)
O4—C10	1.441 (13)	C19—C20	1.393 (15)
O4—C3	1.441 (14)	C19—H19	0.9500
O5—C17	1.452 (12)	C20—C21	1.370 (18)
O5—C6	1.477 (12)	C20—H20	0.9500
O6—C5	1.457 (12)	C21—C22	1.375 (18)
C1—C2	1.533 (16)	C21—H21	0.9500
C1—H1	1.0000	C22—C23	1.375 (14)
C2—C3	1.533 (12)	C22—H22	0.9500
C2—H2	1.0000	C23—H23	0.9500
C3—C4	1.515 (15)	C24—C29	1.375 (15)
C3—H3	1.0000	C24—C25	1.383 (15)
C4—C5	1.521 (12)	C25—C26	1.416 (17)
C4—H4	1.0000	C25—H25	0.9500
C5—C6	1.506 (14)	C26—C27	1.386 (18)
C5—H5	1.0000	C26—H26	0.9500
C6—H6A	0.9900	C27—C28	1.40 (2)
C6—H6B	0.9900	C27—H27	0.9500
C7—C8	1.52 (2)	C28—C29	1.413 (19)
C7—C9	1.515 (19)	C28—H28	0.9500
C8—H8A	0.9800	C29—H29	0.9500
C8—H8B	0.9800	C30—C31	1.394 (15)
C8—H8C	0.9800	C30—C35	1.431 (16)
C9—H9A	0.9800	C31—C32	1.386 (16)
C9—H9B	0.9800	C31—H31	0.9500
C9—H9C	0.9800	C32—C33	1.401 (19)
C10—C11	1.530 (18)	C32—H32	0.9500
C10—H10A	0.9900	C33—C34	1.379 (17)
C10—H10B	0.9900	C33—H33	0.9500
C11—C16	1.35 (2)	C34—C35	1.366 (17)
C11—C12	1.385 (15)	C34—H34	0.9500
C12—C13	1.40 (2)	C35—H35	0.9500
C12—H12	0.9500	C36—H36A	0.9800
C13—C14	1.38 (3)	C36—H36B	0.9800
C13—H13	0.9500	C36—H36C	0.9800
			
O8—S1—O7	117.5 (6)	C14—C13—C12	119.8 (14)
O8—S1—O6	106.9 (5)	C14—C13—H13	120.1
O7—S1—O6	109.3 (4)	C12—C13—H13	120.1
O8—S1—C36	110.3 (10)	C15—C14—C13	121.5 (16)
O7—S1—C36	110.9 (8)	C15—C14—H14	119.3
O6—S1—C36	100.4 (7)	C13—C14—H14	119.3
C1—O1—C4	109.3 (8)	C14—C15—C16	118.8 (18)
C1—O2—C7	109.2 (8)	C14—C15—H15	120.6
C7—O3—C2	105.6 (9)	C16—C15—H15	120.6
C10—O4—C3	113.5 (8)	C11—C16—C15	120.5 (13)
C17—O5—C6	113.2 (8)	C11—C16—H16	119.8
C5—O6—S1	119.7 (7)	C15—C16—H16	119.8
O2—C1—O1	111.2 (10)	O5—C17—C30	110.0 (8)
O2—C1—C2	104.8 (9)	O5—C17—C18	110.0 (8)
O1—C1—C2	107.4 (8)	C30—C17—C18	116.0 (9)
O2—C1—H1	111.1	O5—C17—C24	104.9 (8)
O1—C1—H1	111.1	C30—C17—C24	109.8 (8)
C2—C1—H1	111.1	C18—C17—C24	105.5 (8)
O3—C2—C3	108.8 (9)	C23—C18—C19	117.6 (9)
O3—C2—C1	103.7 (10)	C23—C18—C17	120.3 (10)
C3—C2—C1	103.7 (8)	C19—C18—C17	122.0 (9)
O3—C2—H2	113.3	C18—C19—C20	120.5 (11)
C3—C2—H2	113.3	C18—C19—H19	119.7
C1—C2—H2	113.3	C20—C19—H19	119.7
O4—C3—C4	108.5 (9)	C21—C20—C19	120.5 (12)
O4—C3—C2	110.2 (10)	C21—C20—H20	119.7
C4—C3—C2	101.5 (8)	C19—C20—H20	119.7
O4—C3—H3	112.0	C20—C21—C22	119.4 (10)
C4—C3—H3	112.0	C20—C21—H21	120.3
C2—C3—H3	112.0	C22—C21—H21	120.3
O1—C4—C3	104.4 (8)	C23—C22—C21	119.8 (11)
O1—C4—C5	105.3 (8)	C23—C22—H22	120.1
C3—C4—C5	115.7 (9)	C21—C22—H22	120.1
O1—C4—H4	110.4	C22—C23—C18	122.2 (11)
C3—C4—H4	110.4	C22—C23—H23	118.9
C5—C4—H4	110.4	C18—C23—H23	118.9
O6—C5—C6	112.7 (8)	C29—C24—C25	120.9 (11)
O6—C5—C4	104.7 (8)	C29—C24—C17	120.8 (10)
C6—C5—C4	113.6 (8)	C25—C24—C17	118.3 (9)
O6—C5—H5	108.6	C24—C25—C26	120.4 (10)
C6—C5—H5	108.6	C24—C25—H25	119.8
C4—C5—H5	108.6	C26—C25—H25	119.8
O5—C6—C5	108.8 (9)	C27—C26—C25	119.2 (12)
O5—C6—H6A	109.9	C27—C26—H26	120.4
C5—C6—H6A	109.9	C25—C26—H26	120.4
O5—C6—H6B	109.9	C26—C27—C28	120.0 (13)
C5—C6—H6B	109.9	C26—C27—H27	120.0
H6A—C6—H6B	108.3	C28—C27—H27	120.0
O2—C7—O3	104.9 (8)	C27—C28—C29	120.3 (12)
O2—C7—C8	108.9 (12)	C27—C28—H28	119.8
O3—C7—C8	108.6 (12)	C29—C28—H28	119.8
O2—C7—C9	108.7 (12)	C24—C29—C28	119.2 (12)
O3—C7—C9	112.1 (10)	C24—C29—H29	120.4
C8—C7—C9	113.2 (12)	C28—C29—H29	120.4
C7—C8—H8A	109.5	C31—C30—C35	116.5 (10)
C7—C8—H8B	109.5	C31—C30—C17	120.8 (11)
H8A—C8—H8B	109.5	C35—C30—C17	122.4 (9)
C7—C8—H8C	109.5	C32—C31—C30	120.9 (12)
H8A—C8—H8C	109.5	C32—C31—H31	119.5
H8B—C8—H8C	109.5	C30—C31—H31	119.5
C7—C9—H9A	109.5	C31—C32—C33	121.8 (11)
C7—C9—H9B	109.5	C31—C32—H32	119.1
H9A—C9—H9B	109.5	C33—C32—H32	119.1
C7—C9—H9C	109.5	C34—C33—C32	117.5 (11)
H9A—C9—H9C	109.5	C34—C33—H33	121.3
H9B—C9—H9C	109.5	C32—C33—H33	121.3
O4—C10—C11	108.3 (9)	C35—C34—C33	121.9 (13)
O4—C10—H10A	110.0	C35—C34—H34	119.1
C11—C10—H10A	110.0	C33—C34—H34	119.1
O4—C10—H10B	110.0	C34—C35—C30	121.4 (11)
C11—C10—H10B	110.0	C34—C35—H35	119.3
H10A—C10—H10B	108.4	C30—C35—H35	119.3
C16—C11—C12	121.6 (13)	S1—C36—H36A	109.5
C16—C11—C10	119.8 (10)	S1—C36—H36B	109.5
C12—C11—C10	118.6 (13)	H36A—C36—H36B	109.5
C11—C12—C13	117.9 (16)	S1—C36—H36C	109.5
C11—C12—H12	121.0	H36A—C36—H36C	109.5
C13—C12—H12	121.0	H36B—C36—H36C	109.5
			
O8—S1—O6—C5	−121.0 (9)	C12—C11—C16—C15	2 (2)
O7—S1—O6—C5	7.2 (10)	C10—C11—C16—C15	−178.9 (12)
C36—S1—O6—C5	123.9 (11)	C14—C15—C16—C11	0 (2)
C7—O2—C1—O1	−124.4 (10)	C6—O5—C17—C30	−52.7 (10)
C7—O2—C1—C2	−8.6 (12)	C6—O5—C17—C18	76.3 (10)
C4—O1—C1—O2	98.3 (10)	C6—O5—C17—C24	−170.7 (7)
C4—O1—C1—C2	−15.9 (13)	O5—C17—C18—C23	24.5 (15)
C7—O3—C2—C3	139.1 (9)	C30—C17—C18—C23	150.2 (11)
C7—O3—C2—C1	29.2 (10)	C24—C17—C18—C23	−88.1 (12)
O2—C1—C2—O3	−12.7 (10)	O5—C17—C18—C19	−159.6 (11)
O1—C1—C2—O3	105.7 (10)	C30—C17—C18—C19	−33.9 (16)
O2—C1—C2—C3	−126.4 (9)	C24—C17—C18—C19	87.8 (13)
O1—C1—C2—C3	−7.9 (14)	C23—C18—C19—C20	0 (2)
C10—O4—C3—C4	162.6 (8)	C17—C18—C19—C20	−175.6 (12)
C10—O4—C3—C2	−87.1 (11)	C18—C19—C20—C21	−2 (2)
O3—C2—C3—O4	161.9 (8)	C19—C20—C21—C22	2 (2)
C1—C2—C3—O4	−88.1 (11)	C20—C21—C22—C23	−2 (2)
O3—C2—C3—C4	−83.3 (11)	C21—C22—C23—C18	0 (2)
C1—C2—C3—C4	26.6 (12)	C19—C18—C23—C22	0.3 (19)
C1—O1—C4—C3	33.7 (12)	C17—C18—C23—C22	176.4 (12)
C1—O1—C4—C5	156.0 (9)	O5—C17—C24—C29	−10.1 (12)
O4—C3—C4—O1	79.5 (9)	C30—C17—C24—C29	−128.3 (10)
C2—C3—C4—O1	−36.6 (11)	C18—C17—C24—C29	106.0 (11)
O4—C3—C4—C5	−35.7 (12)	O5—C17—C24—C25	169.0 (8)
C2—C3—C4—C5	−151.8 (10)	C30—C17—C24—C25	50.9 (11)
S1—O6—C5—C6	−75.2 (11)	C18—C17—C24—C25	−74.8 (10)
S1—O6—C5—C4	160.9 (8)	C29—C24—C25—C26	1.3 (15)
O1—C4—C5—O6	−179.3 (9)	C17—C24—C25—C26	−177.9 (9)
C3—C4—C5—O6	−64.6 (12)	C24—C25—C26—C27	−0.5 (15)
O1—C4—C5—C6	57.4 (12)	C25—C26—C27—C28	−1.3 (17)
C3—C4—C5—C6	172.1 (9)	C26—C27—C28—C29	2.3 (19)
C17—O5—C6—C5	−168.5 (7)	C25—C24—C29—C28	−0.3 (17)
O6—C5—C6—O5	−55.3 (11)	C17—C24—C29—C28	178.9 (10)
C4—C5—C6—O5	63.5 (11)	C27—C28—C29—C24	−1.5 (19)
C1—O2—C7—O3	27.1 (13)	O5—C17—C30—C31	−80.3 (13)
C1—O2—C7—C8	143.2 (11)	C18—C17—C30—C31	154.0 (11)
C1—O2—C7—C9	−93.0 (11)	C24—C17—C30—C31	34.7 (14)
C2—O3—C7—O2	−35.1 (12)	O5—C17—C30—C35	94.1 (12)
C2—O3—C7—C8	−151.5 (12)	C18—C17—C30—C35	−31.6 (16)
C2—O3—C7—C9	82.7 (13)	C24—C17—C30—C35	−151.0 (11)
C3—O4—C10—C11	−177.7 (9)	C35—C30—C31—C32	2.3 (19)
O4—C10—C11—C16	−87.3 (13)	C17—C30—C31—C32	177.0 (12)
O4—C10—C11—C12	92.2 (13)	C30—C31—C32—C33	−1 (2)
C16—C11—C12—C13	−2 (2)	C31—C32—C33—C34	−1 (2)
C10—C11—C12—C13	178.8 (12)	C32—C33—C34—C35	0 (2)
C11—C12—C13—C14	0 (2)	C33—C34—C35—C30	1 (2)
C12—C13—C14—C15	1 (3)	C31—C30—C35—C34	−2.6 (19)
C13—C14—C15—C16	−1 (3)	C17—C30—C35—C34	−177.2 (12)

### 1-[(3aR,5R,6S,6aR)-6-Benzyloxy-2,2-dimethyltetrahydrofuro[2,3-d][1,3]dioxol-5-yl]-2-(trityloxy)ethyl methanesulfonate (3) Hydrogen-bond geometry (Å, º)

Cg3 is the centroid of the C11–C16 ring.

**Table:**

*D*—H···*A*	*D*—H	H···*A*	*D*···*A*	*D*—H···*A*
C34—H34···O8^i^	0.95	2.55	3.179 (17)	124
C25—H25···*Cg*3^ii^	0.95	2.99	3.830 (12)	149

Symmetry codes: (i) *x*, *y*−1, *z*; (ii) *x*+1, *y*−1, *z*.

### \ (3aR,5S,6S,6aR)-5-[1-Azido-2-(trityloxy)ethyl]-6-\ benzyloxy-2,2-dimethyltetrahydrofuro[2,3-d][1,3]dioxole (4) Crystal data

**Table:**

C_35_H_35_N_3_O_5_	*D*_x_ = 1.259 Mg m^−^^3^
*M**_r_* = 577.66	Cu *K*α radiation, λ = 1.54178 Å
Orthorhombic, *P*2_1_2_1_2_1_	Cell parameters from 46634 reflections
*a* = 10.0943 (1) Å	θ = 4.3–76.0°
*b* = 10.9625 (1) Å	µ = 0.68 mm^−^^1^
*c* = 27.5392 (2) Å	*T* = 150 K
*V* = 3047.45 (5) Å^3^	Prismatic fragment, colourless
*Z* = 4	0.26 × 0.15 × 0.13 mm
*F*(000) = 1224	

### \ (3aR,5S,6S,6aR)-5-[1-Azido-2-(trityloxy)ethyl]-6-\ benzyloxy-2,2-dimethyltetrahydrofuro[2,3-d][1,3]dioxole (4) Data collection

**Table:**

Agilent SuperNova, Dual, Cu at zero, Atlas diffractometer	6389 independent reflections
Radiation source: SuperNova (Cu) X-ray Source	6282 reflections with *I* > 2σ(*I*)
Mirror monochromator	*R*_int_ = 0.038
Detector resolution: 10.5861 pixels mm^-1^	θ_max_ = 76.5°, θ_min_ = 3.2°
ω scans	*h* = −12→12
Absorption correction: multi-scan (CrysAlis PRO; Rigaku OD, 2015)	*k* = −13→13
*T*_min_ = 0.676, *T*_max_ = 1.000	*l* = −33→34
73112 measured reflections	

### \ (3aR,5S,6S,6aR)-5-[1-Azido-2-(trityloxy)ethyl]-6-\ benzyloxy-2,2-dimethyltetrahydrofuro[2,3-d][1,3]dioxole (4) Refinement

**Table:**

Refinement on *F*^2^	Hydrogen site location: inferred from neighbouring sites
Least-squares matrix: full	H-atom parameters constrained
*R*[*F*^2^ > 2σ(*F*^2^)] = 0.029	*w* = 1/[σ^2^(*F*_o_^2^) + (0.0636*P*)^2^ + 0.2613*P*] where *P* = (*F*_o_^2^ + 2*F*_c_^2^)/3
*wR*(*F*^2^) = 0.085	(Δ/σ)_max_ < 0.001
*S* = 1.03	Δρ_max_ = 0.39 e Å^−^^3^
6389 reflections	Δρ_min_ = −0.18 e Å^−^^3^
391 parameters	Extinction correction: (SHELXL2018; Sheldrick, 2015b), Fc^*^=kFc[1+0.001xFc^2^λ^3^/sin(2θ)]^-1/4^
0 restraints	Extinction coefficient: 0.0007 (2)
Primary atom site location: structure-invariant direct methods	Absolute structure: Flack *x* determined using 2717 quotients [(*I*^+^)-(*I*^-^)]/[(*I*^+^)+(*I*^-^)] (Parsons *et al.*, 2013)
Secondary atom site location: difference Fourier map	Absolute structure parameter: −0.02 (3)

### \ (3aR,5S,6S,6aR)-5-[1-Azido-2-(trityloxy)ethyl]-6-\ benzyloxy-2,2-dimethyltetrahydrofuro[2,3-d][1,3]dioxole (4) Special details

**Table:**

Geometry. All esds (except the esd in the dihedral angle between two l.s. planes) are estimated using the full covariance matrix. The cell esds are taken into account individually in the estimation of esds in distances, angles and torsion angles; correlations between esds in cell parameters are only used when they are defined by crystal symmetry. An approximate (isotropic) treatment of cell esds is used for estimating esds involving l.s. planes.

### \ (3aR,5S,6S,6aR)-5-[1-Azido-2-(trityloxy)ethyl]-6-\ benzyloxy-2,2-dimethyltetrahydrofuro[2,3-d][1,3]dioxole (4) Fractional atomic coordinates and isotropic or equivalent isotropic displacement parameters (Å^2^)

**Table:**

	*x*	*y*	*z*	*U*_iso_*/*U*_eq_	
O1	0.18408 (10)	0.60187 (9)	0.10645 (4)	0.0270 (2)	
O2	0.00591 (11)	0.70764 (11)	0.13994 (4)	0.0328 (3)	
O3	−0.13375 (11)	0.56706 (10)	0.10832 (4)	0.0296 (2)	
O4	0.10606 (11)	0.44527 (11)	0.02350 (4)	0.0291 (2)	
O5	0.15534 (10)	0.23286 (9)	0.13663 (4)	0.0246 (2)	
N1	0.35781 (13)	0.41981 (12)	0.14726 (5)	0.0300 (3)	
N2	0.43200 (15)	0.50814 (14)	0.14439 (5)	0.0354 (3)	
N3	0.5025 (2)	0.58666 (18)	0.14562 (9)	0.0598 (5)	
C1	0.06810 (15)	0.66879 (13)	0.09676 (6)	0.0268 (3)	
H1	0.086933	0.739408	0.074834	0.032*	
C2	−0.03197 (15)	0.58033 (13)	0.07341 (5)	0.0261 (3)	
H2	−0.065704	0.610124	0.041393	0.031*	
C3	0.04319 (15)	0.45956 (13)	0.06926 (5)	0.0248 (3)	
H4	−0.016245	0.388852	0.076452	0.030*	
C4	0.14788 (14)	0.47460 (12)	0.10874 (5)	0.0236 (3)	
H5	0.106924	0.457226	0.141073	0.028*	
C5	0.27373 (14)	0.39970 (13)	0.10348 (5)	0.0246 (3)	
H6	0.323017	0.427614	0.074010	0.030*	
C6	0.24742 (14)	0.26381 (13)	0.09944 (5)	0.0255 (3)	
H6A	0.210161	0.244124	0.067120	0.031*	
H6B	0.330759	0.217442	0.103720	0.031*	
C7	−0.13035 (16)	0.67123 (14)	0.13941 (6)	0.0284 (3)	
C8	−0.16999 (18)	0.6312 (2)	0.18961 (6)	0.0403 (4)	
H8A	−0.261046	0.600252	0.188955	0.060*	
H8B	−0.164574	0.700762	0.211883	0.060*	
H8C	−0.110156	0.566556	0.200704	0.060*	
C9	−0.21657 (19)	0.77199 (16)	0.11883 (7)	0.0397 (4)	
H9A	−0.186880	0.791990	0.085902	0.060*	
H9B	−0.209628	0.844528	0.139474	0.060*	
H9C	−0.308936	0.744573	0.117824	0.060*	
C10	0.01360 (17)	0.42805 (18)	−0.01546 (6)	0.0383 (4)	
H10A	−0.012860	0.508124	−0.028962	0.046*	
H10B	−0.066870	0.386567	−0.003230	0.046*	
C11	0.07737 (15)	0.35211 (16)	−0.05432 (6)	0.0302 (3)	
C12	0.11051 (18)	0.23157 (16)	−0.04514 (6)	0.0354 (4)	
H12	0.093305	0.197681	−0.014029	0.042*	
C13	0.16831 (19)	0.15995 (16)	−0.08068 (7)	0.0382 (4)	
H13	0.190812	0.077529	−0.073932	0.046*	
C14	0.19326 (18)	0.20894 (18)	−0.12618 (6)	0.0374 (4)	
H14	0.233078	0.159988	−0.150614	0.045*	
C15	0.16047 (17)	0.32848 (18)	−0.13608 (6)	0.0370 (4)	
H15	0.177332	0.361637	−0.167347	0.044*	
C16	0.10255 (16)	0.40072 (16)	−0.10021 (6)	0.0338 (3)	
H16	0.080218	0.483138	−0.107031	0.041*	
C17	0.13412 (14)	0.10522 (12)	0.14551 (5)	0.0221 (3)	
C18	0.25661 (14)	0.04939 (14)	0.17016 (5)	0.0247 (3)	
C19	0.33940 (15)	0.12422 (15)	0.19749 (5)	0.0295 (3)	
H19	0.323232	0.209544	0.198606	0.035*	
C20	0.44561 (17)	0.07539 (18)	0.22319 (6)	0.0380 (4)	
H20	0.501120	0.127678	0.241697	0.046*	
C21	0.47113 (18)	−0.04860 (19)	0.22204 (7)	0.0425 (4)	
H21	0.543924	−0.081546	0.239532	0.051*	
C22	0.38886 (18)	−0.12450 (17)	0.19494 (7)	0.0393 (4)	
H22	0.405765	−0.209706	0.193810	0.047*	
C23	0.28199 (16)	−0.07606 (15)	0.16951 (6)	0.0305 (3)	
H23	0.225656	−0.128736	0.151509	0.037*	
C24	0.09417 (14)	0.04392 (13)	0.09745 (5)	0.0239 (3)	
C25	0.18732 (16)	−0.01114 (14)	0.06702 (5)	0.0280 (3)	
H25	0.277293	−0.015847	0.076968	0.034*	
C26	0.14980 (18)	−0.05930 (15)	0.02220 (6)	0.0332 (3)	
H26	0.214126	−0.096942	0.001967	0.040*	
C27	0.0192 (2)	−0.05246 (16)	0.00708 (6)	0.0359 (4)	
H27	−0.006660	−0.087082	−0.023088	0.043*	
C28	−0.07333 (17)	0.00507 (18)	0.03617 (6)	0.0358 (4)	
H28	−0.162552	0.011971	0.025543	0.043*	
C29	−0.03610 (16)	0.05300 (15)	0.08105 (6)	0.0301 (3)	
H29	−0.100456	0.092409	0.100731	0.036*	
C30	0.02126 (14)	0.10232 (13)	0.18324 (5)	0.0231 (3)	
C31	−0.02863 (15)	0.20958 (14)	0.20323 (5)	0.0260 (3)	
H31	0.003067	0.286132	0.191984	0.031*	
C32	−0.12446 (16)	0.20572 (15)	0.23951 (6)	0.0298 (3)	
H32	−0.157422	0.279573	0.252919	0.036*	
C33	−0.17216 (15)	0.09483 (16)	0.25623 (5)	0.0298 (3)	
H33	−0.236550	0.092243	0.281369	0.036*	
C34	−0.12473 (16)	−0.01243 (15)	0.23581 (6)	0.0293 (3)	
H34	−0.158170	−0.088721	0.246647	0.035*	
C35	−0.02869 (15)	−0.00910 (14)	0.19965 (5)	0.0272 (3)	
H35	0.003151	−0.083097	0.186006	0.033*	

### \ (3aR,5S,6S,6aR)-5-[1-Azido-2-(trityloxy)ethyl]-6-\ benzyloxy-2,2-dimethyltetrahydrofuro[2,3-d][1,3]dioxole (4) Atomic displacement parameters (Å^2^)

**Table:**

	*U*^11^	*U*^22^	*U*^33^	*U*^12^	*U*^13^	*U*^23^
O1	0.0230 (5)	0.0237 (5)	0.0343 (5)	−0.0036 (4)	−0.0021 (4)	0.0010 (4)
O2	0.0263 (5)	0.0339 (6)	0.0380 (6)	−0.0031 (5)	−0.0022 (4)	−0.0108 (5)
O3	0.0262 (5)	0.0293 (5)	0.0332 (6)	−0.0044 (4)	0.0042 (4)	−0.0081 (4)
O4	0.0251 (5)	0.0395 (6)	0.0228 (5)	0.0016 (5)	−0.0013 (4)	−0.0039 (4)
O5	0.0245 (5)	0.0235 (5)	0.0259 (5)	0.0000 (4)	0.0055 (4)	0.0011 (4)
N1	0.0248 (6)	0.0338 (7)	0.0314 (7)	−0.0023 (5)	−0.0049 (5)	0.0024 (5)
N2	0.0290 (7)	0.0374 (7)	0.0398 (7)	−0.0013 (6)	−0.0027 (6)	−0.0040 (6)
N3	0.0427 (9)	0.0518 (10)	0.0849 (15)	−0.0151 (9)	−0.0110 (9)	−0.0058 (10)
C1	0.0260 (7)	0.0247 (6)	0.0297 (7)	−0.0020 (5)	−0.0027 (6)	0.0021 (5)
C2	0.0232 (6)	0.0294 (7)	0.0257 (7)	−0.0013 (5)	−0.0015 (5)	−0.0013 (5)
C3	0.0229 (6)	0.0273 (6)	0.0240 (7)	−0.0017 (5)	0.0002 (5)	−0.0024 (5)
C4	0.0233 (6)	0.0233 (6)	0.0242 (6)	−0.0030 (5)	0.0000 (5)	0.0006 (5)
C5	0.0227 (6)	0.0277 (7)	0.0234 (6)	−0.0022 (5)	0.0009 (5)	0.0017 (5)
C6	0.0244 (7)	0.0271 (7)	0.0250 (6)	−0.0003 (5)	0.0048 (5)	0.0016 (5)
C7	0.0255 (7)	0.0280 (7)	0.0317 (7)	−0.0006 (6)	−0.0017 (6)	−0.0057 (6)
C8	0.0345 (8)	0.0554 (10)	0.0309 (8)	0.0002 (8)	0.0004 (7)	−0.0038 (7)
C9	0.0333 (8)	0.0336 (8)	0.0522 (10)	0.0029 (7)	−0.0048 (8)	−0.0029 (7)
C10	0.0307 (8)	0.0541 (10)	0.0299 (8)	0.0096 (8)	−0.0066 (6)	−0.0103 (7)
C11	0.0233 (7)	0.0414 (8)	0.0259 (7)	0.0015 (6)	−0.0049 (5)	−0.0057 (6)
C12	0.0368 (9)	0.0410 (8)	0.0284 (7)	−0.0008 (7)	0.0012 (6)	0.0025 (6)
C13	0.0395 (9)	0.0361 (8)	0.0391 (9)	0.0010 (7)	0.0035 (7)	−0.0033 (7)
C14	0.0302 (8)	0.0480 (10)	0.0339 (8)	−0.0024 (7)	0.0030 (6)	−0.0101 (7)
C15	0.0297 (8)	0.0549 (10)	0.0263 (7)	−0.0035 (7)	0.0007 (6)	0.0039 (7)
C16	0.0277 (7)	0.0407 (8)	0.0331 (8)	−0.0002 (6)	−0.0046 (6)	0.0028 (7)
C17	0.0206 (6)	0.0234 (6)	0.0224 (6)	0.0002 (5)	0.0020 (5)	0.0003 (5)
C18	0.0209 (6)	0.0297 (7)	0.0234 (6)	0.0005 (5)	0.0024 (5)	0.0030 (5)
C19	0.0260 (7)	0.0357 (8)	0.0269 (7)	−0.0015 (6)	−0.0002 (6)	0.0018 (6)
C20	0.0276 (8)	0.0522 (10)	0.0341 (8)	−0.0047 (8)	−0.0062 (6)	0.0052 (7)
C21	0.0279 (8)	0.0562 (11)	0.0433 (9)	0.0059 (8)	−0.0048 (7)	0.0146 (8)
C22	0.0319 (8)	0.0381 (8)	0.0479 (10)	0.0069 (7)	0.0017 (7)	0.0129 (7)
C23	0.0267 (7)	0.0297 (7)	0.0350 (8)	0.0015 (6)	0.0015 (6)	0.0060 (6)
C24	0.0252 (7)	0.0249 (6)	0.0217 (6)	−0.0014 (5)	0.0024 (5)	0.0017 (5)
C25	0.0287 (7)	0.0283 (7)	0.0271 (7)	0.0005 (6)	0.0045 (6)	0.0011 (6)
C26	0.0417 (9)	0.0307 (7)	0.0271 (7)	0.0005 (7)	0.0084 (6)	0.0004 (6)
C27	0.0473 (10)	0.0375 (8)	0.0230 (7)	−0.0076 (7)	−0.0001 (6)	−0.0003 (6)
C28	0.0323 (8)	0.0484 (9)	0.0268 (7)	−0.0053 (7)	−0.0027 (6)	0.0015 (7)
C29	0.0262 (7)	0.0394 (8)	0.0248 (7)	0.0007 (6)	0.0007 (6)	0.0002 (6)
C30	0.0203 (6)	0.0287 (7)	0.0201 (6)	−0.0011 (5)	−0.0008 (5)	0.0003 (5)
C31	0.0253 (7)	0.0284 (7)	0.0243 (6)	−0.0007 (6)	0.0004 (6)	−0.0022 (5)
C32	0.0287 (7)	0.0357 (8)	0.0249 (7)	0.0022 (6)	0.0016 (6)	−0.0065 (6)
C33	0.0248 (7)	0.0444 (8)	0.0201 (6)	−0.0009 (6)	0.0015 (5)	0.0004 (6)
C34	0.0257 (7)	0.0353 (7)	0.0269 (7)	−0.0044 (6)	0.0013 (6)	0.0048 (6)
C35	0.0251 (7)	0.0287 (7)	0.0277 (7)	−0.0013 (6)	0.0022 (6)	0.0008 (6)

### \ (3aR,5S,6S,6aR)-5-[1-Azido-2-(trityloxy)ethyl]-6-\ benzyloxy-2,2-dimethyltetrahydrofuro[2,3-d][1,3]dioxole (4) Geometric parameters (Å, º)

**Table:**

O1—C1	1.4071 (18)	C14—C15	1.379 (3)
O1—C4	1.4437 (16)	C14—H14	0.9500
O2—C1	1.4106 (19)	C15—C16	1.395 (2)
O2—C7	1.4323 (19)	C15—H15	0.9500
O3—C2	1.4146 (18)	C16—H16	0.9500
O3—C7	1.4276 (17)	C17—C24	1.5383 (19)
O4—C3	1.4197 (17)	C17—C18	1.5376 (19)
O4—C10	1.4346 (19)	C17—C30	1.5422 (19)
O5—C6	1.4241 (17)	C18—C19	1.392 (2)
O5—C17	1.4365 (17)	C18—C23	1.399 (2)
N1—N2	1.227 (2)	C19—C20	1.392 (2)
N1—C5	1.4907 (18)	C19—H19	0.9500
N2—N3	1.118 (2)	C20—C21	1.384 (3)
C1—C2	1.541 (2)	C20—H20	0.9500
C1—H1	1.0000	C21—C22	1.392 (3)
C2—C3	1.530 (2)	C21—H21	0.9500
C2—H2	1.0000	C22—C23	1.392 (2)
C3—C4	1.5251 (19)	C22—H22	0.9500
C3—H4	1.0000	C23—H23	0.9500
C4—C5	1.519 (2)	C24—C29	1.394 (2)
C4—H5	1.0000	C24—C25	1.397 (2)
C5—C6	1.517 (2)	C25—C26	1.395 (2)
C5—H6	1.0000	C25—H25	0.9500
C6—H6A	0.9900	C26—C27	1.384 (3)
C6—H6B	0.9900	C26—H26	0.9500
C7—C8	1.505 (2)	C27—C28	1.383 (3)
C7—C9	1.516 (2)	C27—H27	0.9500
C8—H8A	0.9800	C28—C29	1.395 (2)
C8—H8B	0.9800	C28—H28	0.9500
C8—H8C	0.9800	C29—H29	0.9500
C9—H9A	0.9800	C30—C31	1.393 (2)
C9—H9B	0.9800	C30—C35	1.396 (2)
C9—H9C	0.9800	C31—C32	1.391 (2)
C10—C11	1.501 (2)	C31—H31	0.9500
C10—H10A	0.9900	C32—C33	1.386 (2)
C10—H10B	0.9900	C32—H32	0.9500
C11—C12	1.386 (2)	C33—C34	1.388 (2)
C11—C16	1.395 (2)	C33—H33	0.9500
C12—C13	1.384 (2)	C34—C35	1.390 (2)
C12—H12	0.9500	C34—H34	0.9500
C13—C14	1.386 (3)	C35—H35	0.9500
C13—H13	0.9500		
			
C1—O1—C4	107.55 (10)	C12—C13—C14	119.74 (17)
C1—O2—C7	109.54 (11)	C12—C13—H13	120.1
C2—O3—C7	107.93 (11)	C14—C13—H13	120.1
C3—O4—C10	112.81 (12)	C15—C14—C13	120.23 (17)
C6—O5—C17	116.87 (11)	C15—C14—H14	119.9
N2—N1—C5	114.34 (13)	C13—C14—H14	119.9
N3—N2—N1	174.2 (2)	C14—C15—C16	120.01 (16)
O1—C1—O2	111.58 (12)	C14—C15—H15	120.0
O1—C1—C2	107.25 (11)	C16—C15—H15	120.0
O2—C1—C2	104.48 (12)	C15—C16—C11	120.08 (16)
O1—C1—H1	111.1	C15—C16—H16	120.0
O2—C1—H1	111.1	C11—C16—H16	120.0
C2—C1—H1	111.1	O5—C17—C24	108.54 (11)
O3—C2—C3	108.77 (12)	O5—C17—C18	110.07 (11)
O3—C2—C1	104.90 (11)	C24—C17—C18	114.63 (12)
C3—C2—C1	104.51 (12)	O5—C17—C30	104.18 (11)
O3—C2—H2	112.7	C24—C17—C30	112.15 (11)
C3—C2—H2	112.7	C18—C17—C30	106.76 (11)
C1—C2—H2	112.7	C19—C18—C23	118.44 (14)
O4—C3—C4	109.57 (12)	C19—C18—C17	119.13 (13)
O4—C3—C2	112.54 (12)	C23—C18—C17	122.20 (13)
C4—C3—C2	101.35 (11)	C18—C19—C20	120.71 (16)
O4—C3—H4	111.0	C18—C19—H19	119.6
C4—C3—H4	111.0	C20—C19—H19	119.6
C2—C3—H4	111.0	C21—C20—C19	120.64 (17)
O1—C4—C5	107.85 (11)	C21—C20—H20	119.7
O1—C4—C3	104.40 (11)	C19—C20—H20	119.7
C5—C4—C3	116.94 (12)	C20—C21—C22	119.22 (17)
O1—C4—H5	109.1	C20—C21—H21	120.4
C5—C4—H5	109.1	C22—C21—H21	120.4
C3—C4—H5	109.1	C23—C22—C21	120.27 (17)
N1—C5—C4	108.60 (12)	C23—C22—H22	119.9
N1—C5—C6	107.72 (12)	C21—C22—H22	119.9
C4—C5—C6	113.03 (12)	C22—C23—C18	120.71 (16)
N1—C5—H6	109.1	C22—C23—H23	119.6
C4—C5—H6	109.1	C18—C23—H23	119.6
C6—C5—H6	109.1	C29—C24—C25	118.14 (14)
O5—C6—C5	107.18 (12)	C29—C24—C17	119.66 (13)
O5—C6—H6A	110.3	C25—C24—C17	121.91 (13)
C5—C6—H6A	110.3	C24—C25—C26	120.77 (15)
O5—C6—H6B	110.3	C24—C25—H25	119.6
C5—C6—H6B	110.3	C26—C25—H25	119.6
H6A—C6—H6B	108.5	C27—C26—C25	120.28 (15)
O3—C7—O2	104.61 (12)	C27—C26—H26	119.9
O3—C7—C8	108.15 (13)	C25—C26—H26	119.9
O2—C7—C8	109.10 (13)	C28—C27—C26	119.58 (15)
O3—C7—C9	110.17 (13)	C28—C27—H27	120.2
O2—C7—C9	110.61 (13)	C26—C27—H27	120.2
C8—C7—C9	113.78 (15)	C27—C28—C29	120.21 (16)
C7—C8—H8A	109.5	C27—C28—H28	119.9
C7—C8—H8B	109.5	C29—C28—H28	119.9
H8A—C8—H8B	109.5	C28—C29—C24	120.96 (15)
C7—C8—H8C	109.5	C28—C29—H29	119.5
H8A—C8—H8C	109.5	C24—C29—H29	119.5
H8B—C8—H8C	109.5	C31—C30—C35	118.69 (13)
C7—C9—H9A	109.5	C31—C30—C17	121.06 (13)
C7—C9—H9B	109.5	C35—C30—C17	120.18 (13)
H9A—C9—H9B	109.5	C32—C31—C30	120.64 (14)
C7—C9—H9C	109.5	C32—C31—H31	119.7
H9A—C9—H9C	109.5	C30—C31—H31	119.7
H9B—C9—H9C	109.5	C33—C32—C31	120.44 (14)
O4—C10—C11	109.10 (13)	C33—C32—H32	119.8
O4—C10—H10A	109.9	C31—C32—H32	119.8
C11—C10—H10A	109.9	C32—C33—C34	119.23 (14)
O4—C10—H10B	109.9	C32—C33—H33	120.4
C11—C10—H10B	109.9	C34—C33—H33	120.4
H10A—C10—H10B	108.3	C35—C34—C33	120.55 (15)
C12—C11—C16	119.04 (15)	C35—C34—H34	119.7
C12—C11—C10	120.14 (15)	C33—C34—H34	119.7
C16—C11—C10	120.81 (16)	C34—C35—C30	120.43 (15)
C13—C12—C11	120.90 (16)	C34—C35—H35	119.8
C13—C12—H12	119.5	C30—C35—H35	119.8
C11—C12—H12	119.5		
			
C4—O1—C1—O2	92.03 (13)	C10—C11—C16—C15	−179.25 (15)
C4—O1—C1—C2	−21.82 (15)	C6—O5—C17—C24	−54.81 (15)
C7—O2—C1—O1	−127.43 (12)	C6—O5—C17—C18	71.38 (15)
C7—O2—C1—C2	−11.87 (15)	C6—O5—C17—C30	−174.48 (11)
C7—O3—C2—C3	134.00 (12)	O5—C17—C18—C19	25.84 (17)
C7—O3—C2—C1	22.64 (15)	C24—C17—C18—C19	148.52 (13)
O1—C1—C2—O3	111.98 (13)	C30—C17—C18—C19	−86.65 (15)
O2—C1—C2—O3	−6.57 (15)	O5—C17—C18—C23	−159.64 (13)
O1—C1—C2—C3	−2.41 (15)	C24—C17—C18—C23	−36.96 (19)
O2—C1—C2—C3	−120.95 (12)	C30—C17—C18—C23	87.87 (16)
C10—O4—C3—C4	178.79 (13)	C23—C18—C19—C20	0.6 (2)
C10—O4—C3—C2	−69.26 (16)	C17—C18—C19—C20	175.29 (14)
O3—C2—C3—O4	155.11 (12)	C18—C19—C20—C21	0.1 (3)
C1—C2—C3—O4	−93.26 (14)	C19—C20—C21—C22	−0.2 (3)
O3—C2—C3—C4	−87.93 (13)	C20—C21—C22—C23	−0.3 (3)
C1—C2—C3—C4	23.70 (14)	C21—C22—C23—C18	0.9 (3)
C1—O1—C4—C5	162.76 (12)	C19—C18—C23—C22	−1.1 (2)
C1—O1—C4—C3	37.73 (14)	C17—C18—C23—C22	−175.62 (14)
O4—C3—C4—O1	81.90 (13)	O5—C17—C24—C29	−80.11 (16)
C2—C3—C4—O1	−37.20 (13)	C18—C17—C24—C29	156.39 (13)
O4—C3—C4—C5	−37.14 (16)	C30—C17—C24—C29	34.45 (18)
C2—C3—C4—C5	−156.24 (12)	O5—C17—C24—C25	93.58 (15)
N2—N1—C5—C4	−87.25 (16)	C18—C17—C24—C25	−29.91 (19)
N2—N1—C5—C6	150.02 (14)	C30—C17—C24—C25	−151.86 (13)
O1—C4—C5—N1	67.21 (14)	C29—C24—C25—C26	−2.1 (2)
C3—C4—C5—N1	−175.62 (12)	C17—C24—C25—C26	−175.92 (13)
O1—C4—C5—C6	−173.32 (11)	C24—C25—C26—C27	0.4 (2)
C3—C4—C5—C6	−56.15 (16)	C25—C26—C27—C28	1.6 (3)
C17—O5—C6—C5	−168.94 (11)	C26—C27—C28—C29	−1.7 (3)
N1—C5—C6—O5	73.10 (14)	C27—C28—C29—C24	−0.1 (3)
C4—C5—C6—O5	−46.88 (16)	C25—C24—C29—C28	2.0 (2)
C2—O3—C7—O2	−30.07 (16)	C17—C24—C29—C28	175.90 (14)
C2—O3—C7—C8	−146.25 (13)	O5—C17—C30—C31	−4.74 (17)
C2—O3—C7—C9	88.83 (15)	C24—C17—C30—C31	−121.94 (14)
C1—O2—C7—O3	25.84 (16)	C18—C17—C30—C31	111.73 (15)
C1—O2—C7—C8	141.36 (14)	O5—C17—C30—C35	178.22 (12)
C1—O2—C7—C9	−92.76 (15)	C24—C17—C30—C35	61.02 (17)
C3—O4—C10—C11	−150.62 (14)	C18—C17—C30—C35	−65.31 (16)
O4—C10—C11—C12	66.0 (2)	C35—C30—C31—C32	1.3 (2)
O4—C10—C11—C16	−114.86 (17)	C17—C30—C31—C32	−175.82 (13)
C16—C11—C12—C13	0.3 (3)	C30—C31—C32—C33	−0.2 (2)
C10—C11—C12—C13	179.44 (16)	C31—C32—C33—C34	−1.0 (2)
C11—C12—C13—C14	−0.2 (3)	C32—C33—C34—C35	1.2 (2)
C12—C13—C14—C15	−0.1 (3)	C33—C34—C35—C30	−0.2 (2)
C13—C14—C15—C16	0.3 (3)	C31—C30—C35—C34	−1.1 (2)
C14—C15—C16—C11	−0.2 (3)	C17—C30—C35—C34	176.04 (13)
C12—C11—C16—C15	−0.1 (2)		

### \ (3aR,5S,6S,6aR)-5-[1-Azido-2-(trityloxy)ethyl]-6-\ benzyloxy-2,2-dimethyltetrahydrofuro[2,3-d][1,3]dioxole (4) Hydrogen-bond geometry (Å, º)

Cg5 and Cg6 are the centroids of the C24–C29 and C30–C35 rings.

**Table:**

*D*—H···*A*	*D*—H	H···*A*	*D*···*A*	*D*—H···*A*
C13—H13···O3^i^	0.95	2.56	3.281 (2)	133
C1—H1···*Cg*5^ii^	1.00	2.90	3.8014 (16)	150
C8—H8*C*···*Cg*6^iii^	0.98	2.91	3.5435 (18)	123
C12—H12···*Cg*5	0.95	2.88	3.7503 (18)	153
C15—H15···*Cg*6^i^	0.95	2.91	3.6050 (18)	131

Symmetry codes: (i) *x*+1/2, −*y*+1/2, −*z*; (ii) *x*, *y*+1, *z*; (iii) −*x*, *y*+1/2, −*z*+1/2.
